# Cytoreductive treatment in real life: a chart review analysis on 1440 patients with polycythemia vera

**DOI:** 10.1007/s00432-021-03855-5

**Published:** 2021-11-22

**Authors:** Carl C. Crodel, Kathleen Jentsch-Ullrich, Marcel Reiser, Lutz Jacobasch, Annette Sauer, Hans Tesch, Thomas Ulshöfer, Regine Wunschel, Francesca Palandri, Florian H. Heidel

**Affiliations:** 1 Innere Medizin 2, Hämatologie und Onkologie, Universitätsklinikum Jena, Jena, Germany; 2 Gemeinschaftspraxis Hämatologie/Onkologie, Magdeburg, Germany; 3 Pioh Köln Zentrum, Cologne, Germany; 4 Gemeinschaftspraxis Hämatologie und Onkologie, Dresden, Germany; 5 MVZ für Blut—und Krebserkrankungen, Potsdam, Germany; 6 Centrum für Hämatologie und Onkologie, Frankfurt, Germany; 7 Cor/On/Med, Ludwigsburg, Germany; 8 Novartis Pharma GmbH, Nürnberg, Germany; 9 Department of Experimental, Diagnostic and Specialty Medicine, Institute of Hematology L. and A. Seràgnoli, S. Orsola‐Malpighi Hospital, University of Bologna, Bologna, Italy; 10 Innere Medizin C, Hämatologie und Onkologie, Universitätsmedizin Greifswald, Sauerbruchstrasse, 17475 Greifswald, Germany

**Keywords:** Polycythemia vera, PV, Myeloproliferative neoplasia, MPN, Cytoreduction

## Abstract

**Purpose:**

Patients with polycythemia vera (PV) show an elevated incidence of thromboembolic complications and decreased survival when compared to age-matched healthy individuals. Hypercellularity as indicated by elevated hematocrit, pathophysiological changes induced by the *JAK2* driver mutation and cardiovascular risk factors contribute to the increased incidence of thromboembolic events. Higher age and a history of thromboembolic events define a high-risk population of PV patients. Depending on the individual risk profile, phlebotomy or pharmacologic cytoreduction is recommended in combination with low-dose acetylsalicylic acid. Stringent cytoreduction is required for effective risk reduction. However, in recent reports, the rate of thromboembolic complications in PV patients under cytoreductive therapy appears still elevated compared to healthy individuals. This study reports on a chart review to assess for cytoreductive therapy of 1440 PV patients in real life.

**Methods:**

Forty-two eligible hematologists/oncologists in private practice treating patients with MPN were recruited to participate in a paper–pencil-based survey conducted between January 2019 and March 2020 in Germany. Physicians were asked to report primary documented data obtained from patient charts. Descriptive analyses were conducted to assess for patient characteristics, treatment modalities, risk factors and thromboembolic complications.

**Results:**

Data were collected from the patient charts of 1440 individuals diagnosed with PV. The patient population was older than those reported in multicenter trials with a median age of 72.2 years at the time of reporting and 63.5 years at diagnosis. Age was the main factor accounting for high-risk status with 84.7% of patients being above the age of 60 followed by thromboembolic complications reported in 21.3% of patients. The use of pharmacologic cytoreduction was highly variable between participating centers with an average of 60.7% and a range of 10.1–100%. Hydroxyurea was the most frequently used drug followed by ruxolitinib, while interferons were reported for a minority of patients. For 35.4% of patients a persistent need for phlebotomy in addition to cytoreductive treatment was reported. Although presence of high-risk criteria and insufficient disease control were reported as main triggers to initiate pharmacologic cytoreduction, 28.1% had elevated hematocrit values (> 45%) and 38.6% showed persistence of elevated leukocyte count (> 10^9/l^) while on cytoreductive treatment. In contrast, physician-reported symptom burden was lower than published in clinical trials and patient-reported outcomes. The rate of patients experiencing thromboembolic complications was 32.2% at any time and 14.3% after diagnosis with most patients receiving acetylsalicylic acid and 10.8% remaining on oral anticoagulants or heparin.

**Conclusions:**

Cytoreductive treatment of high-risk PV in real life is highly variable regarding indication for cytoreduction and definition of therapy resistance. This study highlights the need for (i) improved risk stratification for thromboembolic events, (ii) consequent indication of pharmacologic cytoreduction in high-risk PV and (iii) attention to signs of therapy resistance that can trigger an earlier and stringent switch to second line agents.

## Introduction

PV is characterized by predominant proliferation of the erythroid lineage, which can be accompanied by hyperplasia of granulopoiesis and megakaryopoiesis. Hyperproliferation of erythroid cells is uncoupled from physiological regulators such as erythropoietin (Epo). The most frequent genetic aberrations in PV are mutations of Janus-kinase 2 (JAK2) (Perner et al. 2019).

Conventional risk stratification in PV is primarily focused on assessing the risk for thromboembolic complications. Nevertheless, the risk for long-term complications, such as leukemia development or myelofibrosis require consideration (Bonicelli et al. 2013; Finazzi and Barbui 2008; Tefferi et al. 2013). Prior history of thrombosis and age are considered the main risk factors for the recurrence of arterial or venous thrombosis (Barbui et al. 2014). More advanced scoring systems include age, history of venous thrombosis and leukocyte counts as established risk factors for survival (Tefferi et al. 2013). The risk for these patients to experience thromboembolic complications is clearly high; however, prognostic parameters beyond age and past medical history of thrombosis are lacking. This leads to challenges in clinical care regarding the indication for and use of cytoreductive drugs and prophylactic treatment with anticoagulants. Most recently, we used a machine learning algorithm to identify risk factors for this high-risk population for clinical use that can predict thromboembolic events (Verstovsek et al. 2019). While prospective validation of these parameters is clearly required, they could be validated in an independent retrospective cohort of PV patients.

First-line treatment for low-risk patients with PV is based on phlebotomies and the use of low-dose ASA, which is a pivotal aspect of PV therapy (Chievitz and Thiede 1962; Lengfelder et al. 2014; Marchioli et al. 2013). In contrast, pharmacologic cytoreduction is recommended for high-risk PV in combination with low-dose ASA therapy (Lengfelder et al. 2014). Cytoreductive drugs should also be considered for low-risk patients who either cannot tolerate phlebotomy, due to high frequency or in case of impending symptomatic iron deficiency. Pharmacologic cytoreduction is also recommended in case of uncontrolled myeloproliferation, characterized by progressive splenomegaly, thrombocytosis or leukocytosis (Barbui et al. 2018; McMullin et al. 2019; Vannucchi et al. 2015a). Cytoreductive agents for clinical use include hydroxyurea (HU) and pegylated interferons (Ropeginterferon-alpha2b) in first line and JAK inhibitors (Ruxolitinib) as a second-line option for HU refractory or intolerant cases.

While multicenter trials investigating therapeutic strategies for PV have been mainly conducted at specialized academic centers, the majority of patients are treated in an outpatient (ambulatory) setting. To assess for the characteristics and treatment modalities in real life we conducted a chart review on cytoreductive treatment modalities.

## Patients and methods

### Aims and objectives

The survey was conducted as a retrospective chart review in primary care centers for patients with hematologic cancers and expertise in the treatment of patients with myeloproliferative neoplasms. Primary goal of this chart review was to assess for frequency, quality and efficacy of cytoreductive therapies in PV patients and for the management of symptoms and complications in this context.

### Recruitment of participants

Recruiting centers have been identified through personal contact and email from a representative panel of board-certified hematologist–oncologists in Germany. Centers that had successfully contributed to previous chart reviews (Jentsch-Ullrich et al. 2016) were also included. As reported before, physicians had to spend more than 50% of their time on patient care. In total, 42 centers participated in this evaluation between January 2019 and March 2020. The documentation was terminated prior to the CoVID-19 pandemic and therefore does not contain any CoVID-19-related changes in symptoms, complications or therapeutic measures. The chart review was performed as a paper–pencil-based questionnaire and documentation was compiled in Excel format. Participating centers received financial compensation for their contributions.

### Questionnaires

Identification of MPN patients with the diagnosis of polycythemia vera (PV) according to the WHO classification 2001, 2008 or 2016 that were documented in the chart review has been conducted in an unbiased manner through the databases of each participating center. The questionnaire was containing questions on (i) patient characteristics, (ii) past medical history and previous therapies, (iii) current laboratory values and molecular data, (iv) disease-related symptoms, (v) current medications and cytoreductive measures and (vi) signs of disease progression.

### Diagnosis, response criteria and risk scores

Diagnosis of PV was confirmed at the participating centers according to the 2001, 2008 or 2016 WHO classifications of myeloid neoplasms (Arber et al. 2016; Barbui et al. 2015b), depending on the date of diagnosis. CTCAE criteria were recommended to report on potential toxicities of cytoreductive measures and the use of ELN criteria were recommended to assess for disease progression (Barosi et al. 2010).

### Ethics, consent and permissions

No identifiable personal information was collected, and the results of the chart review were reported as aggregated datasets of each center. Questionnaires and study materials were reviewed and approved by the institutional review board.

## Results

### Patient characteristics

In total, clinical data on 1440 PV patients were reported by the participating centers. Gender distribution was balanced with 54.3% (*n* = 782) female and 45.7% (*n* = 658) male patients (Table 1). More than half of the patients (62%) had been diagnosed more than 5 years prior to the survey, and the majority (97.3%) had follow-up of more than 1 year after diagnosis. Median age at the time of reporting was 72.2 years and median age at primary diagnosis was 63.5 years. Overall, the patient population was older than in published multicenter trials. 76.7% of patients were above the age of 65 and 84.7% of patients above the age of 60. Thus, age was the main factor accounting for high-risk status.

**Table 1:** Patient characteristics

Characteristics	%	Total *n* = 1440
Sex		
Male	45.7	658
Female	54.3	782
Age (years)		
< 50	4.2	61
50–59	11.0	159
60–64	8.0	115
65–69	11.5	166
≥ 70	65.2	939
Mutation		
JAK2	86.9	1251
JAK2 negative	3.3	47
N/A	9.9	142
Time since diagnosis (years)		
< 1	2.5	36
1–5	35.3	508
6–10	29.1	419
> 10	32.9	474
N/A	0.2	3

**Fig. 1:**
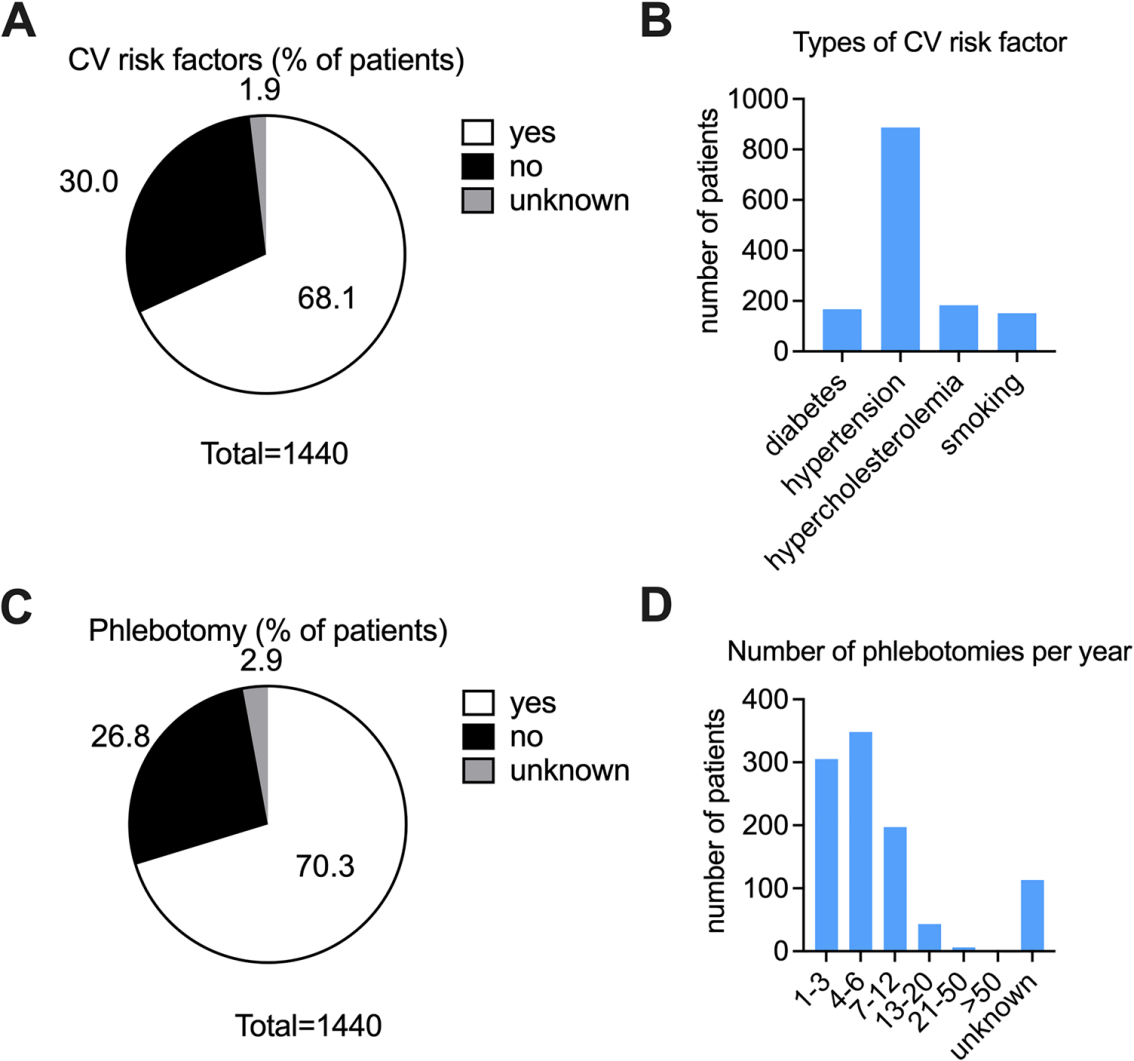
Cardiovascular risk and phlebotomy treatment. **A** Percent of PV patients with additional cardiovascular (CV) risk factors (68.1%, white). In total 1440 patients were analyzed. **B** Type of cardiovascular risk factor and number of patients for each risk factor out of 981patients with at least one CV risk factor. **C** Percent of PV patients treated with phlebotomy (70.3%, white) out of 1440 patients analyzed. **D** Number of patients undergoing phlebotomy treatment, separated by the number of phlebotomies per year (out of *n* = 1012; 70.3%)

Thromboembolic complications were reported in 306 patients (21.3%) at the time of diagnosis and 206 patients (14.3%) had thromboembolic complications after diagnosis and during treatment. 69.2% of patients had no evidence for thromboembolic events. Additional cardiovascular (CV) risk factors were reported by more than two-thirds (68.1%, *n* = 981), whereas 432 patients (30.0%) had no additional risk factors and in 1.9% (*n* = 27) of cases no data was reported. Hypertension was the most prevalent CV risk factor with 90.4% (*n* = 887/981) affected, followed by hypercholesterolemia 18.7% (*n* = 183/981), diabetes mellitus 17.0% (*n* = 167/981) and smoking 15.4% (*n* = 151/981) (Fig. 1A, B). This aspect is of major importance as recent studies have highlighted the impact of comorbidities on the outcome of polycythemia vera patients (Benevolo et al. 2021). Taken together, this data indicates a patient population with a high-risk profile (more than 90% of individuals) regarding thromboembolic complications according to conventional risk stratifications.

### Phlebotomy is the primary measure for cytoreduction in PV

The majority of patients were primarily treated with phlebotomy (70.3%, *n* = 1012, Fig. 1C). On average, a volume of 250–500 ml blood (86.1%, *n* = 871) was drawn and in the majority of cases (71.2%, *n* = 721) the same volume of fluid was substituted intravenously. Oral substitution was used by 16.8% (*n* = 170) of patients. Most patients received less than 1 phlebotomy per month (83.5%). In more detail, 305 PV patients (30.1%) received 1–3 phlebotomies/year, 348 (34.4%) 4–6/year, 197 (19.5%) 7–12 times/year and 49 patients (4.8%) received > 13 phlebotomies/year. For 113 (11.25%) of patients no data for the frequency of phlebotomies was available (Fig. 1D).

Main triggers to proceed to pharmacologic cytoreduction and beyond phlebotomy included the presence of high-risk criteria (36.8%, *n* = 372) and insufficient disease control (41.2%, *n* = 417). Of note, asymptomatic iron deficiency (4.8%, *n* = 49), symptomatic iron deficiency (4.7%, *n* = 48) and intolerance to phlebotomy (2.6%, *n* = 26) have been reported in a relevant number of patients. Also, compliance and patients’ decisions contributed to the limitation of phlebotomy treatment. While this data highlights the importance of phlebotomy as the primary standard of care, its use should be restricted to avoid secondary complications (Heidel et al. 2018) such as iron deficiency or intolerance which affected more than 10% of patients in this real-life setting.

### Use of pharmacologic cytoreduction in real life is highly variable in a high-risk population of PV patients

Next, we assessed for the use of pharmacologic cytoreduction in this high-risk patient population. Pharmacologic cytoreduction is indicated for patients with high-risk PV according to current treatment recommendations (Barosi et al. 2013).

**Fig. 2:**
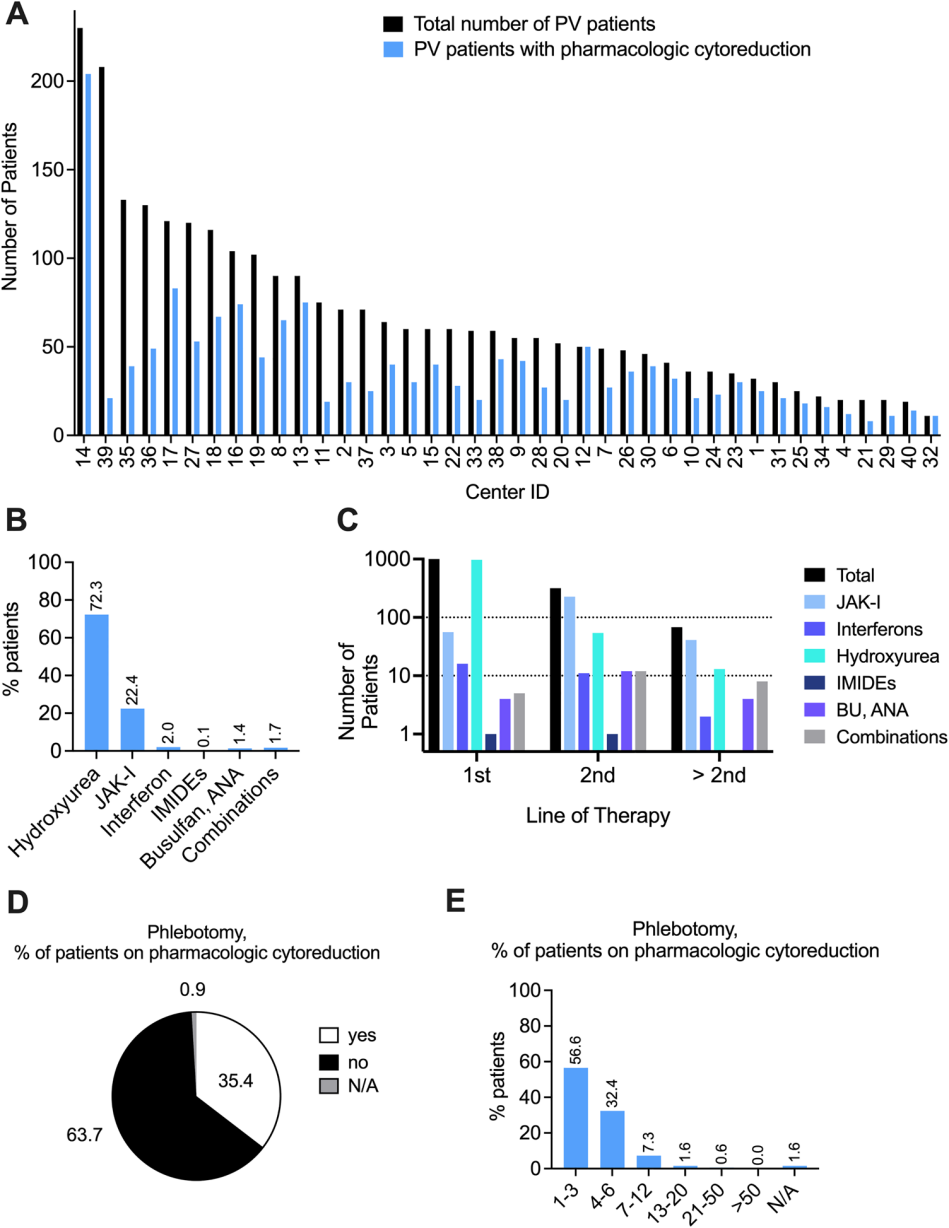
Frequency and characteristics of pharmacologic cytoreduction. **A** Total number of patients per center (black) and absolute numbers of patients per center receiving pharmacologic cytoreduction (blue). **B** Type of pharmacologic cytoreduction, % of patients. **C** Number of patients receiving pharmacologic cytoreduction separated by type of treatment and line of therapy. **D** Percent of patients on phlebotomy treatment while receiving pharmacologic cytoreduction. (E) Percent of patients receiving phlebotomy, separated by the number of phlebotomies per year

Data retrieved from each center was analyzed regarding the total number of PV patients per center (grey bars) compared to those that received pharmacologic cytoreduction (blue bars) (Fig. 2A). Unexpectedly, the fraction of patients treated with pharmacologic cytoreduction was highly variable between the different centers. While the distribution of high-risk patients was comparable between the different investigator sites, the percentage of patients receiving pharmacologic cytoreduction varied between 10.1 and 100%, with an average of 60.7%. When assessing the reasons for lack of pharmacologic cytoreduction in a high-risk patient population among the investigators, patients’ objection to pharmacologic treatment due to potential adverse effects was among the main reasons. Moreover, investigators had chosen not to initiate treatment when low-risk patients reached the age-cutoff of 60 years as the main (or sole) cause of being re-categorized as high risk. However, also patients with cardiovascular risk factors and those diagnosed above the age of 60 were among those remaining exclusively on phlebotomy treatment. These findings indicate that in a real-life setting, a relevant number of patients being at high risk for thromboembolic complications (> 30%) do not receive pharmacologic cytoreduction as recommended by current treatment guidelines (Lengfelder et al. 2018; Vannucchi et al. 2015a).

At the date of exploration (01/2019–03/2020) the predominant choices of cytoreductive agents included hydroxycarbamide/hydroxyurea (HU) (72.3%, *n* = 1041) and JAK1/2-inhibitor ruxolitinib (RUX) (22.4%, *n* = 323) (Fig. 2B). Only a minority of patients received (pegylated) interferons (2.0%, *n* = 29), immunomodulatory drugs (IMIDEs) (0.1%, *n* = 2) or other cytoreductive agents such as busulfan or anagrelide (1.4%, *n* = 20). 25 patients (1.6%) received combinations of cytoreductive agents. While HU was the most frequent cytoreductive agent used in first-line therapy (Fig. 2C), RUX was the most frequent treatment option for second-line treatment or beyond the second line of treatment. Of note, RUX was also used as first-line treatment in 56 patients while only 16 patients started on pegylated interferon (Fig. 2C).

Of note, for 510 (35.4%) patients a persistent need for phlebotomy within the prior 12 months was indicated (Fig. 2D). Of these, 288 (56.5%) received 1–3 phlebotomies/year, 165 (32.4%) 4–6 phlebotomies/year, 37 (7.3%) 7–12 phlebotomies/year and 11 patients (2.2%) received > 13 phlebotomies/year, while for 9 (1.8%) frequency was not reported (Fig. 2E). On average 250-500 ml blood (93.5%, *n* = 77) was drawn and in 83.5% of cases (*n* = 426) the same volume liquid was substituted intravenously. These findings indicate persistent need for phlebotomies in a significant proportion (> 35%) of patients receiving pharmacologic cytoreduction. While phlebotomies lower the risk of thromboembolic complications in low-risk PV (Marchioli et al. 2013), persistent need for phlebotomies during pharmacologic cytoreduction indicates insufficient disease control of the drug treatment. Moreover, risk of thrombosis and HU resistance has been linked with the ongoing need of phlebotomies (Alvarez-Larran et al. 2017). Taken together, low rates of pharmacologic cytoreduction in a high-risk PV population and insufficient disease control by pharmacologic cytoreduction may put this population at risk regarding thromboembolic complications.

### Therapeutic efficacy regarding hematologic parameters and symptom burden

Given the relevant heterogeneity in treatment strategies of this high-risk population as indicated above, we aimed to assess for control of hematologic parameters and disease-associated symptoms. Although repeated measurements of individual hematocrit values were not available due to the nature of the chart review, elevated hematocrit values of > 45% were reported in 28.1% of patients. While 20.3% reported with hematocrit values of 45–48%, 7.8% (*n* = 111) patients had values of 49% or above. These findings indicate that for more than 25% of PV patients on pharmacologic cytoreduction, insufficient control of hematocrit values could be documented. To assess for a second parameter regarding disease control, we analyzed leukocyte counts documented for this patient population. Leukocyte counts are frequently elevated in PV patients and have been verified as a relevant parameter for PV risk stratification (Tefferi et al. 2013). Moreover, several studies have identified an association between leukocytosis and an increased risk of thromboembolic events in patients with PV (Barbui et al. 2015a; De Stefano et al. 2010; Gangat et al. 2007; Lim et al. 2015). In this analysis, 38.6% (*n* = 556) patients showed white blood counts (WBC) of 10^9/l^ or more, while 16.7% (*n* = 241) had an elevated WBC of 15^9/l^ or above upon cytoreductive measures. Persistent leukocytosis despite cytoreductive measures is an indicator of insufficient disease control. In line with the variable number of high-risk PV patients on pharmacologic cytoreduction and the persistent need for phlebotomies in a relevant number of patients, these data indicate lack of stringent cytoreduction according to current treatment recommendations (Barosi et al. 2013).

Beyond the control of hematologic parameters which are indicators for thromboembolic risk, symptom control represents another important therapeutic goal. PV patients suffer from debilitating symptoms irrespective of their risk stratification and therapy, leading to reduced productivity and quality of life (Harrison et al. 2017; Mesa et al. 2016). In this study, classical constitutional symptoms (fever, weight loss, night sweats), general MPN-associated symptoms (fatigue, early satiety, abdominal discomfort, inactivity, problems with concentration, itching, joint/bone pain), splenomegaly and symptoms associated with thrombosis and bleeding were assessed. Moreover, consequences of these symptoms regarding inability to work and reduced working hours were assessed during chart review. Constitutional symptoms were reported in less than 5.5% of patients for all three parameters. While fatigue (20.5%), problems with concentration (20.1%) and inactivity (18.3%) were the most frequently reported general symptoms, the overall symptom burden was modest compared to symptoms reported in published clinical trials. While 8.2% of patients reported microcirculatory symptoms, 4.1% and 3.2% reported on bleeding and signs of thrombosis, respectively. While palpable splenomegaly could be documented in 16.3% of this high-risk PV population, 36.8% of patients could be diagnosed with splenomegaly by sonographic control. Overall, the symptom burden appeared to be moderate compared to symptoms published in previous trials (Vannucchi et al. 2015b) or patient-reported questionnaires (Harrison et al. 2017; Mesa et al. 2016). Consistently, reduced working hours and inability to work were reported at low numbers with 4.8% and 1.5%, respectively. Of note, discrepancies between physician- and patient-reported symptoms have been reported previously.

### Thromboembolic complications

Thromboembolic complications (TEC) represent a major clinical challenge in treatment of PV patients. Patients with high-risk PV show an annual risk for thromboembolic complications of 3.14% compared to 2.23% in low-risk PV, 0.9% in individuals with cardiovascular (CV) risk factors and 0.6% in individuals without CV risk (Alvarez-Larran et al. 2012; Tefferi et al. 2013). Recently published data analyzing risk of thromboembolic events in MPN patients reported that at an early timepoint after diagnosis (3 months) PV patients showed 3- and 13-fold higher risk of arterial and venous thrombosis, respectively, compared with age- and gender-matched controls (Hultcrantz et al. 2018). In previous studies, thromboembolic events have been reported in 12–27% of PV patients and those events were frequently reported in the time before diagnosis or at diagnosis (Polycythemia vera: the natural history of 1213 patients followed for 20 years. Gruppo Italiano Studio Policitemia 1995; Barbui et al. 2014; Cerquozzi et al. 2017; Grunwald et al. 2018; Marchioli et al. 2013).

In our chart review analysis, 32.2% of patients experienced thromboembolic events or major bleeding at any time (before or after diagnosis) and irrespective of concomitant therapy (Fig. 4A). This higher rate of thromboembolic complications can be explained by the high-risk category of PV patients investigated in this analysis. Unfortunately, thromboembolic complications were not stratified by the type of event (arterial versus venous thromboembolism). Consistent with previous reports, the majority of thromboembolic events were reported before diagnosis with 21.3% of patients (Fig. 4B). 14.3% of patients experienced thromboembolic complications after diagnosis (Fig. 4C).

Besides cytoreductive therapy, patients with PV are treated with antiplatelet agents and those with a history of thromboembolic complications are treated with anticoagulants such as oral anticoagulants (OAC, NOAC) or heparins (Griesshammer et al. 2019). Combination of cytoreduction and antiplatelet/anticoagulant therapy has shown an advantageous benefit–risk profile (De Stefano et al. 2018). Regarding antiplatelet therapy, we found 61% of patients receiving documented treatment with acetylsalicylic acid and 2.2% of patients receiving P2Y12 antagonists (e.g. clopidogrel) (Fig. 4D). A total of 10.8% of patients were treated with anticoagulants, such as OACs (4.8%), NOACs (5.7%) and low molecular weight heparins (LMWH, 0.3%). Combinations of antiplatelet agents and anticoagulants were reported in 1.8% of cases. Notably, lack of data was documented in 24.1% of cases (Fig. 4D), which can be explained by the fact that a relevant proportion of patients purchase acetylsalicylic acid products without prescription of their physician. The use of acetylsalicylic acid could be confirmed for the majority of these patients during data discussion among the contributing centers, while a small proportion of patients did not receive antiplatelet therapy due to the use of anagrelide (Fig. 2B). Taking this into consideration, the use of antiplatelet agents was comparable to recently published studies (De Stefano et al. 2018). In contrast, the use of anticoagulants in only 10.8% of patients within a high-risk PV population with 32.2% of patients having experienced thromboembolic complications raised questions about the duration and termination of anticoagulant treatment. While this chart review did not assess for treatment duration and reasons for discontinuation, this data indicates time-restricted use of anticoagulants according to treatment recommendations for non-cancer patients and switch to continued antiplatelet therapy thereafter.

## Discussion

**Fig. 3:**
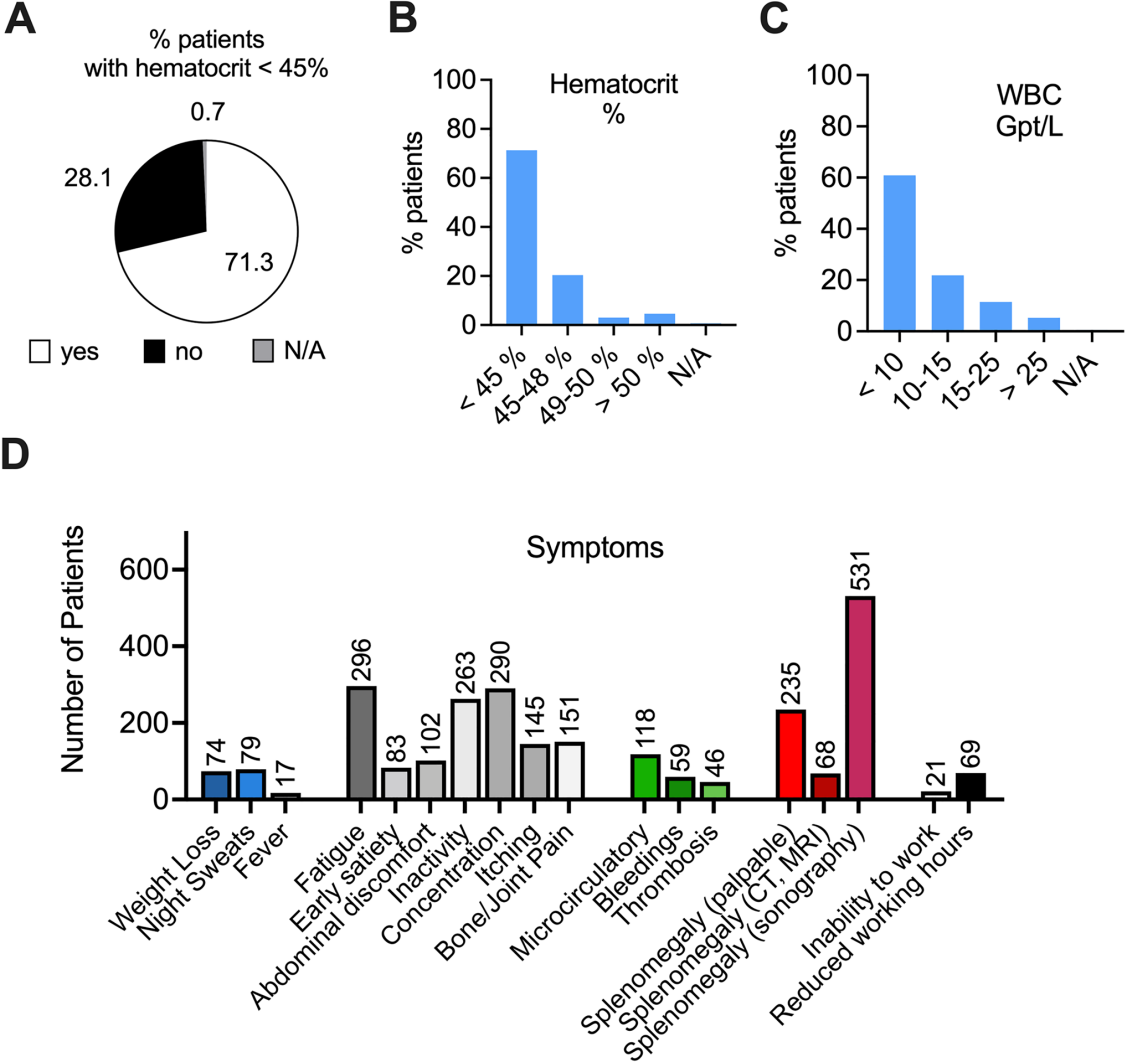
Hematologic parameters and disease-associated symptoms upon cytoreductive treatment. **A** Fraction of patients that achieve hematocrit (Hct) levels below 45%. **B** Fraction of patients separated by hematocrit levels (in %). **C** Fraction of patients separated by leukocyte (WBC) numbers (in Gpt/L). **D** Number of patients that reported constitutional symptoms (blue), general disease-associated symptoms (grey), bleeding or thrombosis (green), splenomegaly (red) and working capability (black) during cytoreductive treatment

Thromboembolic complications (TEC) are the most prevalent clinical challenge in patients with PV (Alvarez-Larran et al. 2017; Griesshammer et al. 2019). Up to 40% of PV patients experience thromboembolic events with arterial and venous thromboses as major determinants of morbidity and mortality. Patients with PV show an annual risk for thromboembolic complications of 3.14% and 2.23% for high-risk and low-risk PV, respectively (Alvarez-Larran et al. 2012; Tefferi et al. 2013). Regarding lethal complications of thromboembolic events in patients with PV an annual rate of 1.1–4.4% was reported (Marchioli et al. 2013; Tefferi 2013). The main therapeutic strategy in patients with PV is risk reduction regarding thromboembolic complications by controlling haematocrit (HCT) to < 45% in combination with antiplatelet therapy. Both measures have led to reduced rates of thromboembolic complications and death (Finazzi et al. 2005; Marchioli et al. 2013). So far, low-risk status is defined by age < 60 years and lack of prior thromboembolic complications. Phlebotomy as a prophylactic measure of risk reduction should result in a rather mild iron deficiency to achieve a state of iron-deficient erythropoiesis and hematocrit control without inducing severe iron-deficiency syndrome. The majority of patients may eventually experience reduction of phlebotomy frequency over time and phlebotomy will be sufficient for hematocrit control without pharmacologic intervention. Progressive splenomegaly, leukocytosis, thrombocytosis, disease-associated symptoms or persistent or high need of phlebotomies may also indicate the need for pharmacologic cytoreduction in low-risk PV patients (Barbui et al. 2011). High-risk patients as defined by age ≥ 60 years and/or with a history of thromboembolic complications (TEC) should be treated with pharmacologic agents for cytoreduction as first-line treatment (Barbui et al. 2018). In this chart review the patient population was of older age compared to published clinical trials (Vannucchi et al. 2015b) and similar to published real life data (Jentsch-Ullrich et al. 2016; Parasuraman et al. 2015). While age was the main factor accounting for high-risk status followed by thromboembolic complications, the vast majority of patients (> 85%) could be categorized as high risk. Unexpectedly and in contrast to published datasets (Benevolo et al. 2021) the indication and use of pharmacologic cytoreduction was highly variable in this chart review. While at least 85% of patients had a clear indication for pharmacologic cytoreduction at the time of analysis, only 60.7% received cytoreductive medication, with a high inter-center variability (10.1–100%). These findings were discussed with investigators from participating center to understand the low number of high-risk patients receiving cytoreductive medication. Among the main reasons for declining pharmacologic cytoreduction, patients’ and physicians’ objection to pharmacologic treatment due to potential adverse effects were reported, especially regarding the use of hydroxyurea and interferons. In Germany, oral hydroxyurea (HU) or subcutaneous, bi-weekly ropeginterferon are the recommended first-line agents, while JAK-inhibitor ruxolitinib is available as a second line option following HU (Lengfelder et al. 2018). However, according to the investigators, the use of ropeginterferon was restricted to a low number of patients due to concerns about price and potential side effects. Moreover, when patients were diagnosed below the age of 60 reached the “age-cutoff” of 60, age was not accepted as a sole trigger to initiate pharmacologic cytoreduction, even in the presence of other cardiovascular risk factors. These findings highlight the need for improved risk stratification using novel biomarkers (Verstovsek et al. 2019) and also to emphasize the proven benefit of pharmacologic cytoreduction. Previous studies had already shown a significant advantage for HU compared to phlebotomy regarding the incidence of cardiovascular events (3.0 versus 5.8/100 person-years, *p* = 0.002) (Barbui et al. 2017). Despite therapy, PV patients on HU still have a significant thrombotic risk (Alvarez-Larran et al. 2016), which can be explained by poorly controlled HCT and persistent need of phlebotomies (Alvarez-Larran et al. 2017), as described in our analysis above (Figs. 1C–D, and 3A–B). Use of HU may be limited by cutaneous toxicity (Griesshammer et al. 2021; Stegelmann et al. 2021) and requires dermatologic controls. Ropeginterferon has been shown as an effective and safe alternative to hydroxyurea also in elderly patients (Gisslinger et al. 2016, 2020) and is approved for first-line treatment in Germany since 2019. Moreover, it can be considered as an effective and safe second-line therapy for patients with PV who are intolerant of or have inadequate response to hydroxyurea. Ropeginterferon has been shown to be effective in inducing hematologic remissions, lowering JAK2-V617F allelic burden, and reducing rates of thrombosis, while discontinuation occurs in a relatively low number of patients (approx. 25%) when compared to early interferon trials (Kiladjian et al. 2006, 2008), HU treatment (Griesshammer et al. 2019) or even ruxolitinib therapy in other subtypes of MPN (Palandri et al. 2020). In our study, the frequent use of ruxolitinib as a second-line option (Fig. 2C) indicates the relevant need to switch from hydroxyurea due to toxicities or insufficient disease and symptom control.

Reduced quality of life due to debilitating symptoms, including pruritus, fatigue and bone pain has been described in recent studies (Harrison et al. 2017; Mesa et al. 2016). According to these studies, disease-associated symptoms impact on the patients’ health, activity and productivity as indicated by reduced working hours in 37% of PV patients and inability to work in 15% (Mesa et al. 2016). In contrast, only 4.8% and 1.5% of patients were reported with reduced working hours and inability to work, respectively, in our physician-reported chart review. This relevant discrepancy can be explained by differences in treatment goals and perception of symptom burden between MPN patients and physicians (Jentsch-Ullrich et al. 2016; Mesa et al. 2017). On the other hand, part of this effect may also be explained in part by the frequent use of ruxolitinib as a second line (> 20%) (or even first line) agent. Ruxolitinib has been shown to be superior to PV standard therapy in controlling hematocrit, reducing spleen size, and improving symptoms associated with polycythemia vera in patients with HU failure (Vannucchi et al. 2015b) and can therefore be considered an effective second line option. Its use may be limited in patients experiencing recurrent infections (Crodel et al. 2020; Landtblom et al. 2020; Polverelli et al. 2015).

**Fig. 4:**
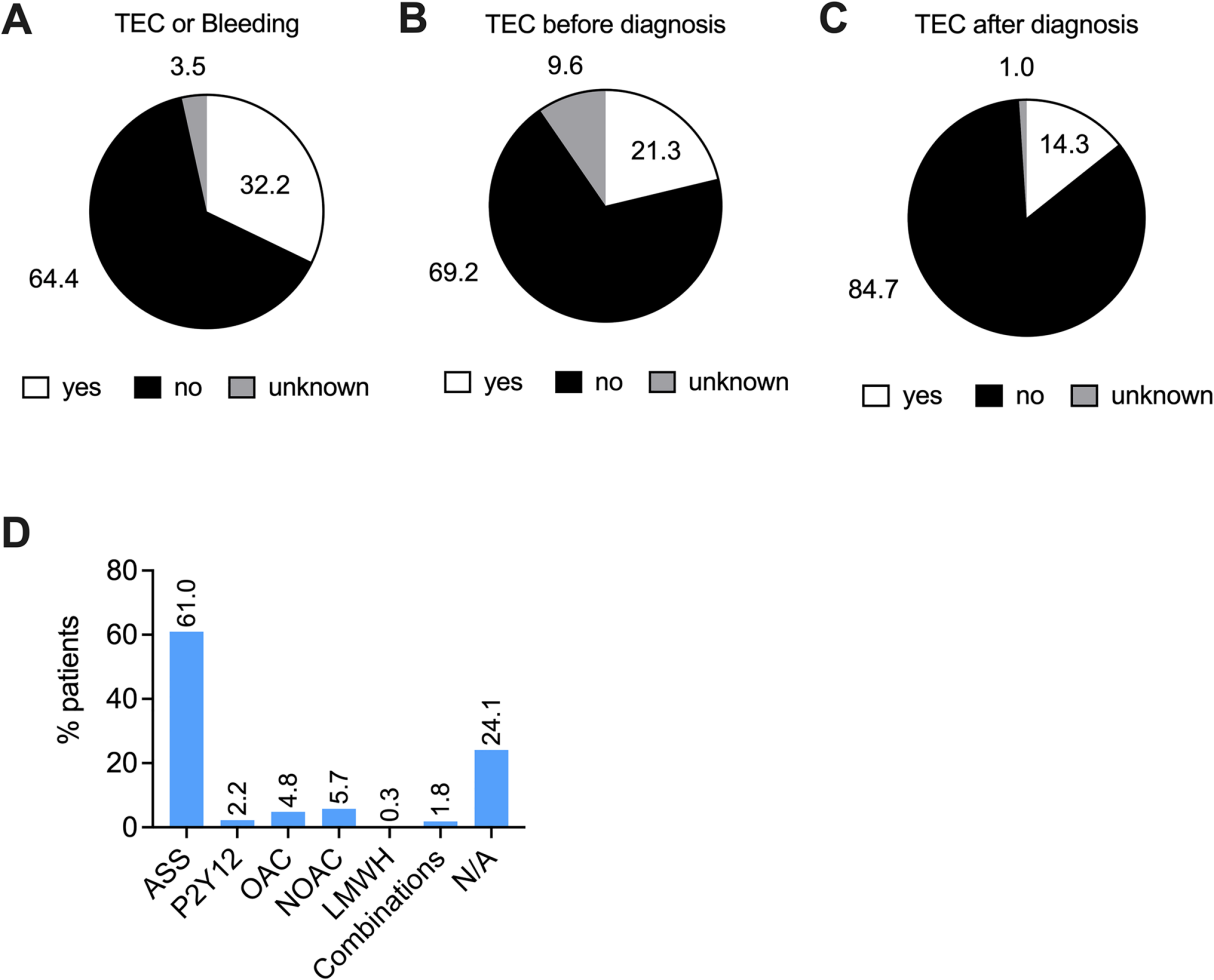
Occurrence of thromboembolic complications (TEC) or severe bleeding events and concomitant use of anticoagulants. **A** Fraction of patients experiencing TEC or bleeding at any time. **B** Fraction of patients with TECs before PV diagnosis (as assessed from past medical history). **C** Fraction of patients with TECs after diagnosis and initiation of PV therapy. **D** Use of anticoagulants indicated as % of patients using either compound

Previous studies have shown that use of acetylsalicylic acid prevented thromboembolic complications (TEC) in PV patients (Landolfi et al. 2004). Use of low-dose acetylsalicylic acid reduced the risk of major thromboembolic complications and death from cardiovascular events (HR 0.40 [95% CI 0.18–0.91] and a *p*-value of 0.03). As reported in this chart review, the majority of patients were either treated with low-dose acetylsalicylic acid (ASS, 100 mg per day) per prescription (61%, Fig. 4D) or by purchasing the drug without prescription (24.1%). In contrast, the low fraction of patients receiving anticoagulants (10.8%) was surprising, given an overall rate of patients with thromboembolic complications of 32.2% (Fig. 4). Of note most patients experienced TECs prior to diagnosis (21.3%), which is in line with published results. 14.3% of patients, however, reported thromboembolic events after diagnosis and during therapy. These findings suggest, that use of anticoagulants was probably time restricted, as recommended for non-cancer patients, followed by switching back on antiplatelet agents. In previous studies, patients treated with oral anticoagulants plus cytoreduction had the lowest rate of recurrences (17.8%) compared with those treated with cytoreduction (50.0%), antiplatelet agents (35.2%), or anticoagulation alone (44.1%) (De Stefano et al. 2008). Follow-up studies reported similar results, with a lower incidence rate of recurrent venous thrombosis in patients receiving oral anticoagulants (4.7% vs 8.9%) (De Stefano et al. 2016). When investigating the duration of treatment these studies found that long-term treatment may reduce the incidence of thromboembolic complications (5.3% versus 12.8%). Potential benefit of a prolonged anticoagulant treatment was recently confirmed by independent groups (Wille et al. 2019) showing recurrent TECs in 36.1% of PV patients who stopped anticoagulant therapy versus 8.6% of patients who continued anticoagulation. Physicians’ recommendations for shortened duration of anticoagulant therapy may arise due to concerns regarding potential bleeding complications. However, in the studies cited above, treatment with anticoagulants did not significantly increase the incidence of major bleeding events, supporting long-term use of oral anticoagulants (OAC or DOAC) in PV patients who have a history of thrombotic events. This is supported by a high safety profile of direct oral anticoagulants (DOAC) in recent studies on MPN and cancer patients (Hamulyak et al. 2021; Huenerbein et al. 2021; Raskob et al. 2018).

In summary, this real life analysis of cytoreductive treatment in a cohort of high-risk PV patients underlines the importance of stringent initiation of pharmacologic cytoreduction according to current treatment guidelines and emphasizes the need for improved risk stratification.
